# Identifying risk factors for early 30-day postoperative complications following pediatric scoliosis surgery: a systematic review and meta-analysis

**DOI:** 10.1007/s43390-026-01351-9

**Published:** 2026-04-01

**Authors:** Sai Sanikommu, Victor M. Lu, Sima M. Vazquez, Amer Samdani, Steven W. Hwang

**Affiliations:** 1 Department of Neurosurgery, Miller School of Medicine, University of Miami, 1501 NW 10th Avenue, 832 G, Miami, FL 33136 USA; 2 Department of Orthopedic Surgery, Shriners Children’s Philadelphia, Philadelphia, PA USA

**Keywords:** Pediatric scoliosis, Postoperative complications, Risk factors, Osteotomy, Pelvic fixation, Perioperative morbidity, Systematic review, Meta-analysis

## Abstract

**Background:**

Early (within 30 days) postoperative complications following pediatric scoliosis surgery continue to be of clinical importance; however, reported risk factors demonstrate inconsistency across various studies. We conducted a systematic review and meta-analysis to quantify patient- and procedure-related predictors of 30-day postoperative outcomes complications.

**Methods:**

A systematic search of PubMed, Embase, Scopus, and Web of Science identified observational studies reporting risk factors for 30-day postoperative complications following scoliosis surgery. Adjusted odds ratios (ORs) with 95% confidence intervals (CIs) were pooled using random-effects models. Heterogeneity was assessed with the *I*^2^ statistic.

**Results:**

Ten observational studies comprising 113,082 patients met the inclusion criteria; nine reported adjusted estimates. Osteotomy was significantly associated with increased risk (OR = 1.40; 95% CI 1.25–1.55; *I*^2^ = 0%), as was pelvic fixation (OR = 1.65; 95% CI 1.07–2.55; *I*^2^ = 0%), with no observed heterogeneity across studies. In contrast, multiple medical and patient-related risk factors including cardiopulmonary, hematologic and metabolic comorbidities were not significantly associated with postoperative morbidity, although point estimates suggested a directionally elevated risk and were characterized by wide confidence intervals. Male sex and neuromuscular disorders were also not associated with a significant increase in risk.

**Conclusions:**

Among the evaluated factors, complex surgical techniques involving osteotomy and pelvic fixation were the most consistent predictors of early postoperative complications within the contemporary literature. Preoperative patient comorbidities showed variable, generally non-significant associations, underscoring the need for standardized risk definitions and prospective multicenter studies to better predict outcomes in the early postoperative period.

**Supplementary Information:**

The online version contains supplementary material available at https://doi.org/10.1007/s43390-026-01351-9.

## Introduction

Pediatric scoliosis surgery is associated with a meaningful risk of early postoperative morbidity, with reported 30-day complication rates ranging widely across institutions and patient populations [1–6]. While advances in surgical technique, perioperative monitoring, and anesthesia have improved overall safety, postoperative complications, including pulmonary, infectious, and resource-intensive events, remain a significant source of morbidity, prolonged hospitalization, and healthcare utilization [5, 7–9]. Identifying patients at elevated risk of early postoperative complications is therefore critical for informing preoperative counseling, perioperative optimization, and risk-adjusted benchmarking.

Numerous observational studies have examined patient and procedure-related risk factors for early postoperative complications following pediatric scoliosis surgery, including comorbid cardiopulmonary disease, obesity, neuromuscular etiology, curve severity, and operative complexity [1–6, 8, 10–13]. However, the existing literature is highly fragmented, with individual studies variably reporting associations between the same risk factors and different postoperative outcomes [2, 10, 11]. As a result, clinicians must rely on isolated single-center estimates, often derived from heterogeneous definitions of complications and limited sample sizes, making it difficult to determine which risk factors demonstrate consistent and clinically meaningful associations across studies [14, 15]. This heterogeneity has limited cross-study comparability and precluded the clear identification of risk factors demonstrating consistent, reproducible associations with early postoperative morbidity [16]. Consequently, clinical decision-making continues to rely on isolated single-center estimates or registry-based analyses that may be underpowered for specific outcomes, susceptible to residual confounding, and difficult to generalize across practice settings.

To date, no comprehensive quantitative synthesis has systematically evaluated risk factors for 30-day postoperative complications in pediatric scoliosis surgery while accounting for outcome-specific reporting and methodological heterogeneity. Prior reviews have been narrative or focused on selected complications, without formal pooling of effect estimates. In this study, we conducted a systematic review and meta-analysis, in accordance with PRISMA guidelines, to synthesize the available evidence on risk factors for early 30-day postoperative complications in pediatric scoliosis surgery. Meta-analyses were performed where sufficient data were available using outcome-specific pooling to avoid unit-of-analysis errors, with structured narrative synthesis employed when quantitative pooling was not feasible.

## Methods

### Protocol and guidance

This systematic review and meta-analysis was conducted in accordance with the Preferred Reporting Items for Systematic Reviews and Meta-Analyses (PRISMA) reporting guideline [17]. The study protocol and methods were registered with PROSPERO a priori (1277935).

### Search strategy and eligibility criteria

A systematic literature search was conducted across multiple databases including the Ovid Embase, PubMed, Scopus, Web of Science, and Cochrane Central Library. Searches encompassed records from database inception through December 2025 and were adapted to the indexing and search syntax of each database. The complete electronic search strategy for all databases are detailed in Supplementary Table 1.

Studies were eligible if they included pediatric patients (≤ 18 years) undergoing surgical treatment for scoliosis and reported postoperative complications within 30 days of surgery in association with patient, disease, or procedure-related risk factors. Studies were required to report effect estimates as adjusted odds ratios (ORs) with corresponding confidence intervals. Studies enrolling fewer than 20 patients, those limited to adult populations, studies reporting only late postoperative outcomes without separable early complication data, and non-original research articles were excluded. Detailed inclusion and exclusion criteria are provided in Supplementary Table 2.

The primary outcome was defined a priori as the occurrence of any postoperative complication within 30 days of surgery, as reported by the individual studies. This composite endpoint encompassed medical, surgical, infectious, pulmonary, and resource-related complications. Medical complications were defined as systemic or organ-specific adverse events not directly attributable to the surgical construct (e.g., cardiac events, thromboembolism, urinary tract infection). Surgical complications included events directly related to the operative procedure or instrumentation (e.g., surgical site infection, wound dehiscence, implant-related complications, neurologic injury, or reoperation). Pulmonary complications were categorized separately and included respiratory failure, pneumonia, and prolonged ventilatory support. Resource-related complications referred to outcomes reflecting increased healthcare utilization, such as prolonged length of stay, unplanned intensive care unit admission, or 30-day readmission. A single composite outcome was selected to enhance comparability across studies and to avoid unit-of-analysis errors arising from overlapping reporting of multiple complication subtypes within the same patient cohorts.

To facilitate quantitative synthesis across studies that variably reported comorbidities and patient characteristics, we organized related predictors into clinically coherent physiologic domains prior to pooling. This domain-based approach was chosen to improve interpretability and feasibility of meta-analysis in the setting of heterogeneous reporting, while aligning groupings with shared mechanisms relevant to early postoperative morbidity after pediatric deformity surgery. Specifically, anthropometric factors (e.g., age, BMI) were grouped because body habitus and developmental/physiologic reserve have been repeatedly associated with perioperative risk and complications in pediatric and spine surgery populations [13]. Metabolic factors (e.g., diabetes, hypertension) were grouped given their established links to systemic dysregulation, microvascular impairment, and postoperative complications in surgical populations, and because large database studies in scoliosis surgery commonly treat these as comorbidity-risk constructs rather than isolated exposures [5, 13]. Cardiopulmonary factors (e.g., asthma, cardiomyopathy/heart failure) were grouped because impaired ventilatory/hemodynamic reserve is directly relevant to postoperative pulmonary and systemic complications after scoliosis surgery, and these conditions have been associated with increased complication risk in pediatric posterior spinal fusion cohorts [18]. We acknowledge that domain-level pooling may introduce conceptual heterogeneity and may mask variable-specific effects; therefore, domain estimates are interpreted as indicators of broader physiologic vulnerability rather than equivalence among individual predictors.

### Study selection and data collection process

The search strategy was systematically implemented across all databases. Retrieved records were first processed using EndNote X9, where duplicate entries were identified and removed. The remaining records were then exported in.XML format to Mendeley for further organization. Mendeley was used to convert the data into RIS format, allowing for expert review through the Rayyan platform (https://www.rayyan.ai/). Duplicate articles were carefully removed, and an initial screening of titles and abstracts was conducted by two independent reviewers (SS, SMV) based on predefined inclusion criteria to identify relevant studies. Discrepancies among reviewers were initially resolved through discussion, with a third reviewer (VML) serving as an arbitrator in cases where consensus could not be reached.

Data extraction was performed independently by two reviewers (SS and SMV) using a standardized data collection form. Extracted data included study characteristics, patient demographics, scoliosis etiology, surgical details when available, and evaluated risk factors. When studies reported both unadjusted and adjusted estimates, adjusted estimates were preferentially extracted. Any discrepancies in data extraction were resolved through discussion and consensus.

### Risk of bias and certainty of evidence

The quality of the studies included in this analysis was evaluated based on the guidelines outlined in the Cochrane reviewers’ handbook [19]. For the methodological assessment of non-randomized studies, two independent reviewers (SS and SMV) utilized the Risk of Bias in Non-randomized Studies of Interventions (ROBINS-I) tool [20].

To evaluate the certainty of the evidence derived from the eligible studies, a quantitative synthesis was conducted following the Cochrane recommendations [19]. The grading of recommendation, assessment, development, and evaluation (GRADE) approach was employed.

### Data synthesis

A meta-analysis was conducted when at least two studies reported the same effect estimate for a specific outcome. Given the limited number of studies contributing to individual analyses, for the meta-analysis, we computed pooled adjusted ORs with their 95% confidence intervals (CIs) for all outcomes using a random-effects model with the Hartung–Knapp adjustments. For risk factors reported by fewer than two studies, findings were summarized using structured narrative synthesis without quantitative pooling. Formal assessment of publication bias was not performed due to the small number of studies per analysis. We utilized the Cochran *Q* and *I*^2^ tests to evaluate statistical heterogeneity. An *I*^2^ statistic with values of > 25%, > 50%, and > 75% indicated low, considerable, and substantial levels of heterogeneity, respectively. [21] All statistical analyses were conducted using R software (version 4.2.0).

## Results

### Study characteristics

**Fig. 1:**
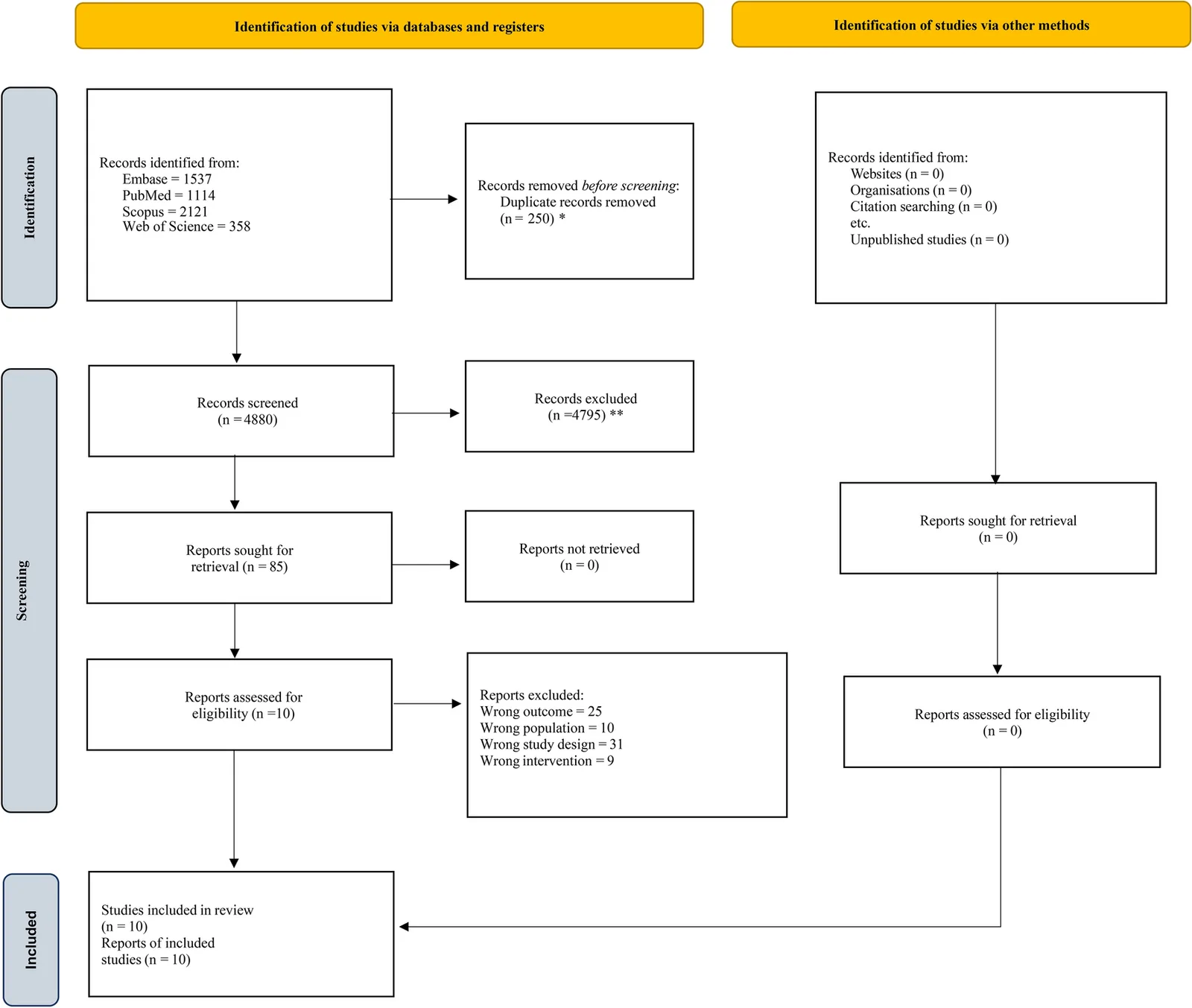
PRISMA flowchart of study selection. * With automation tool; ** Without automation tool. Wrong intervention: studies not evaluating surgical correction of pediatric scoliosis (e.g., nonoperative treatment, adult deformity cohorts). Wrong outcome: studies not reporting early (≤ 30-day) postoperative complications or lacking extractable complication-related data. Wrong population: studies reporting data of adults or mixed populations Wrong study design: studies that do not report regression analysis, case-series, reviews, and editorial

After screening titles and abstracts from 5130 records, 85 full-text articles were assessed for eligibility. Ten observational studies met the inclusion criteria, comprising a total of 113,082 patients [1, 3–6, 8, 10–13]. Among the included studies, nine provided adjusted ORs for risk factors associated with 30-day postoperative complications [1, 3–6, 8, 11–13], and one study mentioned number and percentages [10]. Seven studies (70%) are from the USA [1, 3–6, 10, 11] and three (30%) [8, 12, 13] from the rest of the world. Three studies (30%) reported the outcomes among idiopathic scoliosis^1,3,13^, four studies (40%) are non-specific [4, 5, 10, 11], one study (10%) reported outcomes of early-onset scoliosis [12], and Milak et al. studied outcomes for both idiopathic and neuromuscular scoliosis separately [6], giving rise to two effect sizes from the same study (Table 1 and Fig. 1).

**Table 1:** Characteristics of studies assessing risk factors for postoperative 30-day complications in pediatric patients undergoing surgery for scoliosis correction

Author (Year)	Country	Study period	Age groups	No. of patients	Female	Etiology of scoliosis	Risk factors evaluated	Risk factors included in meta-analysis	Outcomes reported	Adjusted covariates
McKee et al. (2018)	USA	2012–2015	< 6 to 18	7089	5395	Idiopathic Scoliosis	Congenital heart disease and Cardiomyopathy	Congenital heart disease and cardiomyopathy	Mortality, Any Complication, SSI	Age, sex, race, BMI-for-age percentile, ASA status, number of levels fused, perioperative transfusion, neuromuscular comorbidity, and operative time
Goheer et al. (2025)	USA	2016–2021	0–18	32,621	22,276	NA	Developmental delay	Developmental delay	Mortality, SSI, Medical Complications	Age, sex, race, admission status, surgical approach, scoliosis type, ventilator dependence, pulmonary and cardiac comorbidities, neurologic comorbidities, nutritional status, hematologic disorders, ASA class, fusion type, transfusion, and operative time (model selection via AIC-based stepwise regression)
Elsamadicy et al. (2021)	USA	2016–2018	10–18	4929	3603	Idiopathic Scoliosis	Age, Anemia, Gastric and Pulmonary disease	Hematological disorder (Anemia), Age	Mortality, SSI, Medical Complications	Age, anemia, structural pulmonary/airway abnormalities, esophageal/gastrointestinal disease, and number of levels fused
Deveza et al. (2022)	USA	July 2014 to June 2019	0–18	240	124	Congenital, congenital scoliosis; NMS, neuromuscular scoliosis; SS, syndromic scoliosis	Congenital, congenital scoliosis; Neuromuscular scoliosis; Syndromic scoliosis ± Pelvis fixation, Pelvis fixation	Pelvis fixation	SSI, Readmission in 30 days and Reoperation in 90 days and revision surgery in 1 year	BMI, pulmonary disease, technology dependence, seizure disorder, number of levels fused, and pelvis fixation
Reames et al. (2011)	USA	2004–2007	0 to 18	19,360	NA	Idiopathic, Congenital, Neuromuscular, Others	Idiopathic, Congenital, Neuromuscular, Others	NA	Any Complication, Mortality, SSI, Pulmonary complications	NA
Taniguchi et al. (2018)	Japan	2010–2013	0–18	1703	1357	NA	Age, Sex, Diabetes, Allogenic blood transfusions	Age, Male sex, Diabetes	Any complication	Male sex, diabetes, and allogeneic blood transfusion
Ramos et al. (2016)	USA	2002–2011	10–18	36,355	NA	Idiopathic Scoliosis	Age, Male sex, Cardiopulmonary risk factors (heart Failure), Metabolic disorder (HTN, DM), Osteotomy, Obesity, Renal failure, Perivascular disorder, Hypothyroidism, Drug abuse, Procedure, No of levels fused Approach	Age, Male sex, Cardiopulmonary risk factors (Heart Failure), Metabolic disorder (HTN, DM), Osteotomy	Any Complication	Age, sex, primary payer, anemia, heart failure, drug abuse, hypertension, hypothyroidism, obesity, revision procedure, surgical approach, and osteotomy (stepwise multiple logistic regression; variables entered if p < 0.15
Zhang et al. (2022)	China	2013–2018	NA	73	43	Early-onset	Cardiopulmonary risk factor, Etiology, Pre transferrin	Cardiopulmonary risk factor	Pulmonary complication	Blood loss/total blood volume ratio ≥ 15%, pulmonary comorbidities, pretransferrin < 200 mg/dL, and scoliosis etiology (non-idiopathic vs idiopathic)
Al Lede et al. (2020)	Australia	2009–2012	2–17	133	90	NA	Age, Pre surgical respiratory volumes	Age	Any Complication	Age and FEV1% predicted (backward logistic regression; NIV use and CO₂ retention evaluated but not retained)
Malik et al. (2019)	USA	2012–2016	0–18	10,579	7765	Neuromuscular and Idiopathic	Cardiopulmonary risk factor (Asthma), Developmental delay, Osteotomy, Pelvic fixation, Hematological disorder, Neuromuscular disorder, Race, Ventilator dependence, Gastric disease, no of levels fused	Cardiopulmonary risk factor (Asthma), Developmental delay, Osteotomy, Pelvic fixation, Hematological disorder, Neuromuscular disorder	Pneumonia, Mortality, Cardiac Complications	Age, race, asthma, ventilator dependence, gastric disease, developmental delay/impaired cognition, neuromuscular disorders, hematologic disorder, inotropic support, number of levels fused, osteotomy, pelvic fixation, and intervertebral device insertion (separate models for idiopathic and neuromuscular scoliosis)

*DM* diabetes mellitus, *HTN* hypertension

Four studies (40%) reported a relation between anthropometric risk factors and postoperative 30-day complications [4, 5, 8, 13], four studies (40%) assessed cardiopulmonary risk factors [1, 6, 12, 13], three studies reported hematological risk factors [3, 6, 13], two studies each reported a relation between developmental delay [6, 11], male sex [5, 13], metabolic disorders [5, 13], osteotomy [5, 13]and pelvic fixation [4, 6]. Malik et al. studied the association between neuromuscular disorders, hematological disorders, osteotomy, pelvic fixation, and developmental delay and postoperative 30-day complications in idiopathic and neuromuscular disorders, respectively; we treated these as two distinct cohorts for our analysis (Table 2).

**Table 2:** Summary of pooled associations between evaluated risk factors and 30-day postoperative complications following pediatric scoliosis surgery

Risk factor	No. of studies	Effect estimates	OR (95% CI)	*I*^*2*^	Grade
Anthropometric	4	4	(OR = 0.90; 95% CI [0.79–1.02])	71.8%	⨁⨁◯◯ Low
Cardiopulmonary risk factors	4	6	(OR = 2.55; 95%CI [0.77–8.41])	79.0%	⨁◯◯◯ Very low
Male sex	2	2	(OR = 1.83; 95% CI [0.77–4.39])	0.0%	⨁⨁◯◯ Low
Developmental delay	2	3	(OR = 1.43; 95% CI [0.89–2.28])	54.6%	⨁◯◯◯ Very low
Hematological disorder	3	4	(OR = 1.74; 95%CI [0.96–3.16])	50.4%	⨁⨁◯◯ Low
Metabolic disorder	2	3	(OR = 2.94; 95% CI [0.39–22.32])	44.9%	⨁◯◯◯ Very low
Neuromuscular disorder	1	2	(OR = 1.30; 95% CI [0.62–2.74])	0.0%	⨁◯◯◯ Very low
Osteotomy	2	3	(OR = 1.40; 95% CI [1.25–1.55])	0.0%	⨁◯◯◯ Very low
Pelvic fixation	2	3	(OR = 1.65; 95% CI [1.07–2.55])	0.0%	⨁◯◯◯ Very low

For each risk factor, the number of contributing studies and effect estimates are shown, along with pooled odds ratios (ORs) and 95% confidence intervals (CIs) derived from random-effects meta-analyses. Between-study heterogeneity was assessed using the *I*^2^ statistic. Certainty of evidence was graded according to the GRADE framework for observational studies, with downgrades primarily based on risk of bias (ROBINS-I), inconsistency, and imprecision

### Anthropometric measures and 30-day post-operative complications

**Fig. 2:**
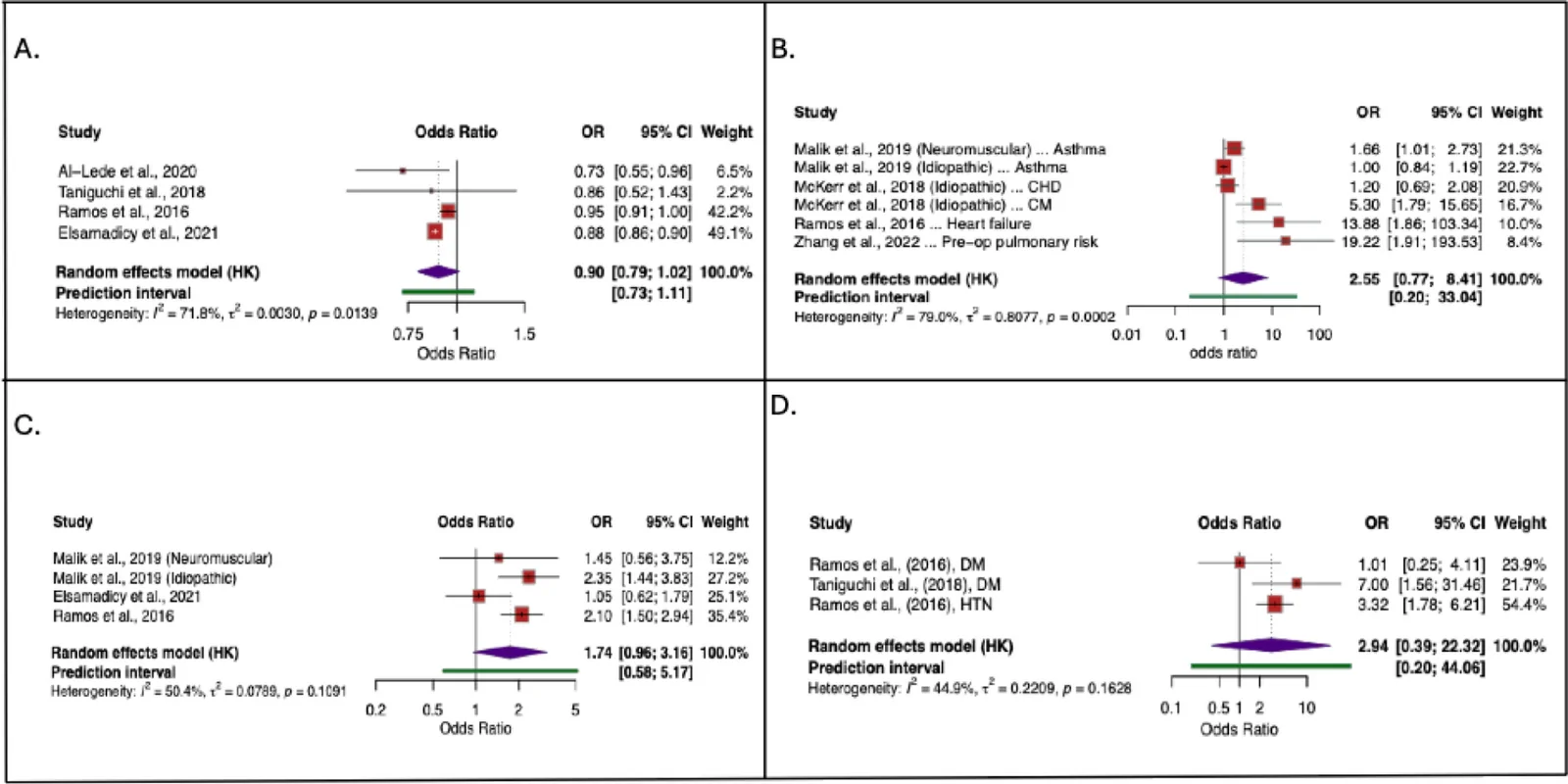
Pooled associations between patient comorbidity categories and 30-day postoperative complications. Forest plots summarize study-level adjusted odds ratios (red squares) with corresponding 95% confidence intervals (horizontal lines) for four predefined risk-factor domains: **A** Anthropometric factors, **B** cardiopulmonary disorders, **C** hematologic disorders, and **D** metabolic disorders. The size of each square is proportional to the inverse variance (study weight)

Pooled analyses demonstrated no consistent association between anthropometric factors and 30-day postoperative complications following pediatric scoliosis surgery (OR = 0.90; 95% CI [0.79–1.02], *I*^*2*^ = 71.8%), across five estimates evaluating age and BMI (Fig. 2A).

### Cardiopulmonary risk factors and 30-day post-operative complications

Cardiopulmonary risk factors, including asthma, congenital heart disease, cardiomyopathy, heart failure, and composite preoperative pulmonary risk, were reported in five studies, yielding six effect estimates. In the overall pooled analysis, these factors were associated with a non-significantly higher estimate for any 30-day postoperative complication (OR = 2.55; 95%CI [0.77–8.41], *I*^*2*^ = 79%) (Fig. 2B).

Subgroup analyses demonstrated similar non-significant patterns. In the cardiac comorbidity subgroup, pooled estimates suggested increased odds of postoperative complications (OR = 3.56; 95%CI [0.18–71.99], *I*^*2*^ = 79.8%). In the pulmonary risk subgroup, which included asthma and composite preoperative pulmonary risk, pooled estimates again showed increased odds of postoperative complications (OR = 2.03; 95%CI [0.08–48.44], *I*^*2*^ = 79.1%) (Supplementary Fig. 1A and B). The wide CIs reflect considerable imprecision across studies.

### Hematological disorders and 30-day post-operative complications

Hematological disorders, including anemia, were identified in three studies, contributing to four effect estimates. In the aggregate analysis, these factors demonstrated a non-significant tendency toward an increased risk of any 30-day postoperative complication (OR = 1.74; 95%CI [0.96–3.16], *I*^*2*^ = 50.4%) (Fig. 2C).

### Metabolic disorders and 30-day post-operative complications

Metabolic disorders, encompassing hypertension and diabetes, were documented in two studies, resulting in three effect estimates. In the combined analysis, these factors demonstrated a non-significant trend toward an increased risk of any 30-day postoperative complication (OR = 2.94; 95% CI [0.39–22.32], *I*^*2*^ = 44.9%) (Fig. 2D).

### Developmental delay and 30-day post-operative complications

**Fig. 3:**
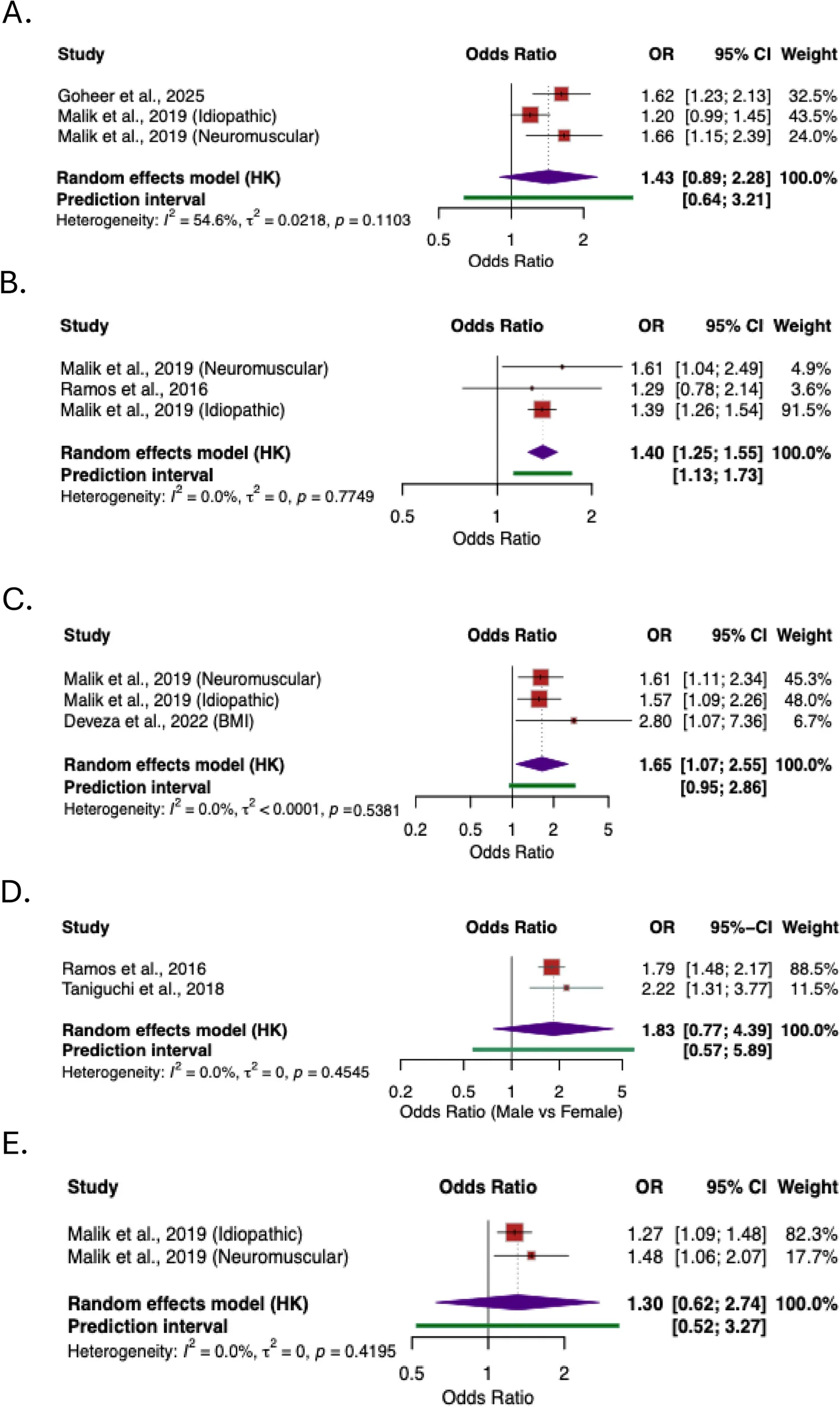
Pooled associations of selected patient- and procedure-related factors with 30-day postoperative complications. Forest plots show study-level adjusted odds ratios with corresponding 95% confidence intervals for the following factors: **A** Developmental delay, **B** osteotomy, **C** pelvic fixation, **D** male sex, and **E** neuromuscular disorders

Developmental delay exhibited a non-significant correlation with an elevated risk of any 30-day postoperative complication in pediatric patients undergoing scoliosis surgery (OR = 1.43; 95% CI [0.89–2.28], *I*^*2*^ = 54.6%) (Fig. 3A).

### Surgical approach and 30-day post-operative complications

Among surgical approaches, osteotomy (OR = 1.40; 95% CI [1.25–1.55], *I*^*2*^ = 0%) and pelvic fixation (OR = 1.65; 95% CI [1.07–2.55], *I*^*2*^ = 0%) were shown to significantly increase the risk for postoperative 30-day complications among pediatric patients undergoing surgery for scoliosis (Fig. 3B and C).

### Male sex, neuromuscular disorders, and 30-day post-operative complications

Male sex was associated with a higher likelihood of postoperative complications within 30 days (OR = 1.83; 95% CI [0.77–4.39], *I*^*2*^ = 0%); however, this increase did not achieve statistical significance. Associated neuromuscular disorders showed the same trend with non-significantly increased odds for postoperative complications within 30 days (OR = 1.30; 95% CI [0.62–2.74], *I*^*2*^ = 0%) (Fig. 3D and E).

### Risk of bias and certainty of evidence

Overall, all included studies were observational in design and demonstrated varying risk of bias, as assessed using the ROBINS-I tool. Most studies were judged to have low to moderate risk of bias, while several registry-based analyses exhibited serious to considerable risk due to residual confounding and limitations inherent to administrative data sources (Supplementary Fig. 2).

In the GRADE assessment, the certainty of evidence ranged from low to very low across the evaluated risk factors, with downgrades primarily driven by risk of bias, inconsistency across studies, and imprecision in effect estimates. No concerns regarding indirectness or publication bias were identified (Table 2 and Supplementary Table 3).

## Discussion

This systematic review and meta-analysis of 10 observational studies, encompassing more than 113,000 pediatric patients undergoing scoliosis surgery, examined patient-related, comorbidity-related, and operative risk factors associated with any postoperative complication within 30 days. While numerous patient-level risk factors have been proposed in previous research, our pooled analyses revealed that most such factors, including anthropometric measures, cardiopulmonary disease, metabolic disorders, hematologic conditions, developmental delay, sex, and neuromuscular etiology, were not consistently linked to early postoperative complications and exhibited considerable heterogeneity and imprecision. Conversely, operative factors indicative of increased surgical complexity, specifically osteotomy and pelvic fixation, demonstrated reproducible and statistically significant associations with early postoperative morbidity. Collectively, these findings suggest that procedure-related factors may have a greater and more consistent impact on early postoperative outcomes than baseline patient characteristics in pediatric scoliosis surgery.

The lack of strong correlations for most patient-level risk factors is probably indicative of both biological and methodological influences [22]. Pediatric scoliosis populations are inherently diverse, encompassing idiopathic, neuromuscular, syndromic, and early-onset etiologies, each exhibiting unique physiological reserves, comorbidity profiles, and perioperative management approaches [10, 23]. Many clinically relevant factors contributing to postoperative risk, such as curve rigidity, pulmonary function testing, nutritional status, functional impairment, and perioperative optimization, are measured variably or incompletely documented in registry and administrative datasets [22, 24–26].

Consequently, patient-related risk factors may show apparent associations in single center or disease-specific cohorts but may not demonstrate consistent effects across varied populations^10,13^. Similar variability has been observed in prior large registry-based studies, including the Scoliosis Research Society Morbidity and Mortality Database and the Nationwide Inpatient Sample, in which the direction and strength of associations for comorbidities such as asthma, obesity, and metabolic disease varied considerably depending on outcome definitions and adjustment methods [10, 13, 24].

Conversely, operative complexity has emerged as a more dependable determinant of early postoperative risk [27, 28]. Both osteotomy and pelvic fixation were consistently correlated with elevated odds of 30-day complications and exhibited no observable heterogeneity across studies. However, these pooled estimates were derived from a limited number of contributing studies and should therefore be interpreted with appropriate caution. These findings align with clinical intuition, as such procedures are recognized to increase operative duration, blood loss, physiological stress, and postoperative care requirements [10, 28, 29]. Prior research in pediatric and adult spinal deformity surgery has consistently demonstrated that increasing surgical invasiveness, including three-column osteotomies and pelvic fixation constructs, is associated with higher rates of early complications, resource utilization, and reoperation [10, 29, 30]. However, it is important to note that the included studies in the present meta-analysis did not consistently differentiate between specific osteotomy techniques (e.g., posterior column osteotomy, pedicle subtraction osteotomy, or vertebral column resection). In most cases, osteotomy was reported as a binary procedural variable within large database analyses, limiting the ability to assess differential risk across specific osteotomy subtypes. Importantly, these operative strategies are frequently employed in patients with greater curve severity, neuromuscular or syndromic etiologies, and increased baseline complexity, and therefore, the observed associations may partly reflect confounding by indication rather than independent causal effects, despite multivariable adjustment.

Our findings further reinforce the notion that the extent of surgical intervention may be a more actionable predictor of early postoperative morbidity than static patient characteristics, particularly in pediatric populations, where baseline health status is often relatively preserved. Although osteotomy and pelvic fixation demonstrated statistically significant associations with early postoperative complications, these findings were derived from a limited number of contributing studies and should therefore be interpreted cautiously. Moreover, these operative techniques are frequently employed in patients with greater curve severity, neuromuscular or syndromic etiologies, and increased baseline complexity. As such, residual confounding by indication cannot be excluded, even in studies that adjusted for baseline characteristics. These procedures may therefore function as markers of surgical and disease complexity rather than purely independent causal determinants of postoperative morbidity.

Although we preferentially extracted the most fully adjusted estimates, covariate selection varied across studies. Differences in adjustment strategy may introduce residual confounding if key baseline or operative factors were not included, or overadjustment if mediators were controlled for. Therefore, pooled adjusted effects should be interpreted as associative rather than strictly causal estimates.

From a methodological perspective, the overall low to very low certainty of evidence identified through the GRADE approach emphasizes significant limitations within the existing literature. The majority of included studies were observational and susceptible to residual confounding, while heterogeneity in the definitions of complications impeded comparability across studies. Additionally, substantial statistical heterogeneity observed in certain subgroup analyses particularly within cardiopulmonary and anthropometric domains (*I*^2^ ≈ 80%) likely reflects between-study differences in scoliosis etiology (idiopathic versus neuromuscular), variation in surgical complexity and fusion extent, institutional case volume, and differences in perioperative optimization protocols. The extremely wide CIs further indicate imprecision and limited stability of pooled estimates in these domains. Accordingly, these subgroup findings should be interpreted as exploratory rather than definitive risk magnitudes. Taken together, the risk of bias, inconsistency, and imprecision identified through GRADE limit the strength of inferences that can be drawn, and firm conclusions regarding the independent contribution of specific risk factors remain constrained by the current evidence base.

These results underscore the necessity for standardized reporting of early postoperative complications, harmonized definitions of risk factors, and enhanced documentation of disease severity and functional status in future multicenter registries. At a minimum, future studies should incorporate clearly defined 30-day complication criteria; standardized classification of scoliosis etiology (idiopathic, neuromuscular, congenital, syndromic); objective measures of baseline curve severity and functional status; detailed reporting of operative variables including fusion length, osteotomy type, and pelvic fixation; and transparent multivariable adjustment strategies specifying included confounders. Until such data are available, caution should be exercised when applying individual patient-level predictors to perioperative counseling or risk stratification models. Conversely, our findings advocate for a greater focus on operative planning, procedural complexity, and modifiable surgical factors when evaluating early postoperative risk in pediatric scoliosis surgery.

### Limitations

This study presents several limitations that merit careful consideration. First, all included studies employed observational designs, rendering the pooled estimates vulnerable to residual confounding despite the preferential extraction of adjusted effect sizes. Critical clinical variables, such as curve rigidity, pulmonary function testing, nutritional status, functional impairment, perioperative optimization strategies, and surgeon- or center-level factors, were inconsistently reported and could not be adjusted for uniformly across studies. Objective measures of surgical center volume, surgeon experience, and detailed indices of deformity severity were likewise inconsistently available, limiting the ability to evaluate potential volume–outcome relationships or hierarchical confounding in the observed associations between procedural complexity and postoperative morbidity. Second, the definitions of postoperative complications varied considerably, with some studies reporting composite outcomes and others concentrating on specific complication subtypes. This variability limits the comparability across studies and contributes to heterogeneity. In particular, the use of a composite “any complication” endpoint—while methodologically necessary to enhance cross-study comparability—limits clinical granularity and precluded reliable stratified meta-analyses evaluating whether operative factors such as osteotomy or pelvic fixation differentially influence specific complication subtypes (e.g., infectious, pulmonary, or neurological events). Third, several risk factors were examined in a limited number of studies, resulting in imprecise effect estimates with wide CIs and low certainty of evidence. Additionally, most included studies combined mixed scoliosis etiologies without reporting stratified adjusted estimates, precluding reliable etiology-specific subgroup meta-analyses and potentially introducing clinical heterogeneity. Finally, the reliance on registry and administrative databases in multiple included studies introduces potential misclassification and coding biases. These limitations highlight the necessity for standardized outcome definitions and harmonized reporting of risk factors in future multicenter pediatric spine research.

## Conclusion

This systematic review and meta-analysis suggests that current evidence does not consistently demonstrate statistically robust or high-certainty associations between most patient-level comorbidity factors and 30-day postoperative complications following pediatric scoliosis surgery. However, the presence of wide confidence intervals, substantial heterogeneity in certain domains, and overall low certainty of evidence indicate imprecision rather than a definitive absence of effect. Operative factors reflecting increased surgical complexity, such as osteotomy and pelvic fixation, were associated with higher rates of early postoperative morbidity. However, these findings should be interpreted with caution, as they may partly reflect procedural complexity and confounding by indication rather than independent causal effects. These findings suggest that procedure-related factors may play an important role in early postoperative risk, although the contribution of baseline patient characteristics remains uncertain and warrants further high-quality investigation. Future research should aim to standardize the definitions of early complications, improve the assessment of disease severity and functional status, and harmonize adjustment strategies to enable more precise perioperative risk stratification and informed clinical decision-making in pediatric scoliosis surgery.

## Supplementary Information

Below is the link to the electronic supplementary material.

Supplementary file1 (DOCX 309 kb)

## Data Availability

The datasets generated during and/or analyzed during the current study are available from the corresponding author on reasonable request.
