# Supplementary material for: Identifying risk factors for early 30-day postoperative complications following pediatric scoliosis surgery: a systematic review and meta-analysis
Source: Spine Deform. 2026 Apr 1;14(5):1827–39. doi: 10.1007/s43390-026-01351-9 (PMC13553619; doi:10.1007/s43390-026-01351-9)
Supplement: Supplementary file 1 [file 43390_2026_1351_MOESM1_ESM.docx]

**Supplementary Table 1.** Prisma Search strategy

| Database | Search terms | No of studies |
| --- | --- | --- |
| PubMed | scoliosis OR "spinal deformity" OR kyphoscoliosis OR "neuromuscular scoliosis")  AND  ("spinal fusion" OR "spine surgery" OR instrumentation OR arthrodesis)  AND  (pediatric OR paediatric OR child OR children OR adolescent)  AND  (mortality OR death OR complication OR "postoperative complication" OR "respiratory failure" OR pneumonia OR sepsis)  AND  ("risk factor" OR predictor OR association OR regression)  NOT  (adult) | 1114 |
| Embase | (scoliosis OR spinal deformity OR kyphoscoliosis OR neuromuscular scoliosis)  AND  (spinal fusion OR spine surgery OR instrumentation OR arthrodesis)  AND  (pediatric OR paediatric OR child OR children OR adolescent)  AND  (mortality OR death OR complication OR postoperative complication OR respiratory failure OR pneumonia OR sepsis)  AND  (risk factor OR predictor OR association OR regression)  NOT  (adult) | 1537 |
| Scopus | (scoliosis OR "spinal deformity" OR kyphoscoliosis OR "neuromuscular scoliosis")  AND  ("spinal fusion" OR "spine surgery" OR instrumentation OR arthrodesis)  AND  (pediatric OR paediatric OR child OR children OR adolescent)  AND  (mortality OR death OR complication OR "postoperative complication" OR "respiratory failure" OR pneumonia OR sepsis)  AND  ("risk factor" OR predictor OR regression)  AND NOT  (adult) | 2121 |
| Web of Science | (scoliosis OR "spinal deformity" OR kyphoscoliosis OR "neuromuscular scoliosis")  AND  ("spinal fusion" OR "spine surgery" OR instrumentation OR arthrodesis)  AND  (pediatric OR paediatric OR child OR children OR adolescent)  AND  (mortality OR death OR complication OR "postoperative complication" OR "respiratory failure" OR pneumonia OR sepsis)  AND  ("risk factor" OR predictor OR regression)  NOT  (adult) | 358 |

**Supplementary Table 2.** Inclusion and Exclusion Criteria of Studies.

| **Inclusion criteria** | **Exclusion criteria** |
| --- | --- |
| - Pediatric patients (typically ≤18 years) undergoing **scoliosis corrective surgery** (posterior fusion/instrumentation ± anterior approach; deformity correction) - Any scoliosis etiology (idiopathic, neuromuscular, congenital, syndromic, early-onset, mixed), provided scoliosis surgery population is clear - Studies assessing **risk factors/predictors** for postoperative outcomes (demographics, comorbidities, anthropometrics, etiology, surgical/technical factors, pre-op status) - **Reporting 30-day postoperative complications** (any complication, medical complications, SSI, pulmonary/cardiac complications) - Provides **adjusted effect estimates** (aOR/OR/RR/HR) with 95% CI - Observational analytic studies: retrospective/prospective cohorts, registry/administrative database studies, case–control - Any publication year (from database inception to search end date) - English (or languages you can reliably extract/translate, if applicable) - Any country/health system | - Adults only; mixed adult/pediatric cohorts without separable pediatric data; non-scoliosis spinal surgery only - Non-scoliosis deformities only (e.g., kyphosis-only) unless scoliosis data separable - Studies reporting outcomes without evaluating predictors (descriptive series only), unless raw 2×2 data allow effect estimation - Outcomes not within 30 days (e.g., 90-day, 1-year) without separable 30-day data; purely radiographic outcomes (curve correction only), PROMs only - No comparator and no computable effect size (e.g., complication rate only in one subgroup with no reference group); duplicate reporting of the same cohort without unique data - Case reports, small case series without comparisons (e.g., <10 patients), editorials, narrative reviews, conference abstracts without full data - Non-English if full-text data cannot be extracted |

| **Outcome** | **Study design** | **Risk of bias** | **Inconsistency** | **Indirectness** | **Imprecision** | **Publication bias** | **Overall certainty** |
| --- | --- | --- | --- | --- | --- | --- | --- |
| **Cardiopulmonary disease** (asthma, CHD, cardiomyopathy, HF, pre-op pulmonary risk) | Observational (registry / cohort) | Serious | Moderate-high | No | Serious | Undetected | ⨁◯◯◯ Very Low |
| **Anthropometric** | Observational | Moderate | Low | No | Not Serious | Undetected | ⨁⨁◯◯ Low |
| **Sex** (Male) | Observational | Low | Low | No | Not Serious | Undetected | ⨁⨁◯◯ Low |
| **Neuromuscular disorders** | Observational | Low | Moderate | No | Not Serious | Undetected | ⨁◯◯◯ Very Low |
| **Hematologic disorders** | Observational | Moderate | Low | No | Not Serious | Undetected | ⨁⨁◯◯ Low |
| **Developmental delay** | Observational | Moderate | Moderate | No | Serious | Undetected | ⨁◯◯◯ Very Low |
| **Metabolic disorders (**DM, HTN) | Observational | Considerable | High | No | Serious | Undetected | ⨁◯◯◯ Very Low |
| **Pelvic fixation** | Observational | Low | Moderate | No | Not Serious | Undetected | ⨁◯◯◯ Very Low |
| **Osteotomy** | Observational | Low | Moderate | No | Not Serious | Undetected | ⨁◯◯◯ Very Low |

**Supplementary Table 3.** GRADE assessment of certainty of evidence for associations between evaluated risk factors and **30-day postoperative complications** following pediatric scoliosis surgery. All included studies were observational. Certainty ratings were determined using the GRADE framework and reflect downgrades for risk of bias (assessed with ROBINS-I), inconsistency, and imprecision. Indirectness and publication bias were not identified across outcomes.

**Supplementary Figure 1.** Forest plots showing the association between cardio-pulmonary comorbidities and 30-day postoperative complications following pediatric scoliosis surgery. (A) Cardiac comorbidity subgroup, including congenital heart disease (CHD), cardiomyopathy (CM), and heart failure. (B) Pulmonary risk subgroup, including asthma and composite preoperative pulmonary risk. Pooled odds ratios (ORs) with 95% confidence intervals (CIs) were calculated using random-effects models with Hartung–Knapp adjustment. Between-study heterogeneity is reported using the I² statistic.

**Supplementary Figure 2. Risk of Bias assessment using the ROBINS-I tool.**
